# Editorial: Looking for a culprit: The role of environmental co-factors in complex neuropsychiatric disorders

**DOI:** 10.3389/fnins.2022.1000783

**Published:** 2022-08-24

**Authors:** Mirko Manchia, Clement C. Zai, Daniela Fanni, Gavino Faa

**Affiliations:** ^1^Section of Psychiatry, Department of Medical Sciences and Public Health, University of Cagliari, Cagliari, Italy; ^2^Unit of Clinical Psychiatry, University Hospital Agency of Cagliari, Cagliari, Italy; ^3^Department of Pharmacology, Dalhousie University, Halifax, NS, Canada; ^4^Tanenbaum Centre for Pharmacogenetics, Campbell Family Mental Health Research Institute, Centre for Addiction and Mental Health, Toronto, ON, Canada; ^5^Department of Psychiatry, Institute of Medical Science, Laboratory Medicine and Pathobiology, University of Toronto, Toronto, ON, Canada; ^6^Unit of Anatomic Pathology, Department of Medical Sciences and Public Health, University of Cagliari, Cagliari, Italy; ^7^Unit of Anatomic Pathology, University Hospital Agency of Cagliari, Cagliari, Italy

**Keywords:** omics, epigenetics, genomics, risk prediction, severe mental disorders

There is consensus that complex disorders, including most neuropsychiatric and somatic disorders, have etiological diatheses determined by the interaction of genetic architecture with environmental triggers (Plomin et al., 2009; Tenesa and Haley, 2013). Depression, diabetes, asthma, hepatic tumors, renal and cardiovascular disease are just some of the many conditions where environmental factors modulate the liability conferred by genetic factors. While on the one hand there have been advances in the quantification of the risk conferred by genetic factors with the advent of genome-wide association studies (Plomin and von Stumm, 2022), on the other hand there is less certainty on the extent to which environmental factors might modulate the liability threshold toward disease. In cancer, epidemiological evidence showed ample differences in incidence between populations, with large changes in incidence occurring over a relatively short period or on migration, constituting the most pertinent evidence for environmental causation of cancer (Hemminki et al., 2006). This has led to the development of methods for the assessment of cumulative exposure to environmental triggers of disease (Hu et al., 2021). Indeed, Hu et al. (2021) established a cutting-edge method, based on gas chromatography high-resolution mass spectrometry, to operationalize the human exposome and provide a standardized method to obtain quantitative information on known and/or unidentified environmental chemicals that might influence the illness risk. Further, in schizophrenia, a psychiatric disorder with an heritability estimate of about 80%, recent evidence showed the possibility of quantifying, with relatively good accuracy, the environmental loading of a range of exposures (Pries et al., 2019). Specifically, environmental risk factors explained up to 20% of its heritability (or phenotypic variance) (Pries et al., 2019). Another recent study showed that heritability estimates under a model containing the full repertoire of environmental and gene–environment interaction terms was 46%, indicating a substantial contribution of the exposome on the risk of developing schizophrenia (Zhang et al., 2022). Of note, neurological disorders such as Parkinson's disease, where the heritability is typically small, have a more distinct role of environmental triggers as modulators of disease risk. This points to a clear, although still not precisely quantified, role of environmental determinants in the development of complex disorders. Another important question that remains partly unanswered is why these complex disorders still show epidemic aspects, with raising incidence observed over the last decades despite the continuous progress in prevention, diagnosis, and treatment. This conundrum might be partly explained by the theory of decanalization (Gibson, 2009). Accordingly, the rapid evolution of the human genome, combined with marked environmental and cultural perturbations in the past two generations, might have led to the uncovering of cryptic genetic variations that represent a major source of disease susceptibility and explain the increased susceptibility toward complex disorders (Gibson, 2009). In this context, our Research Topic aimed to gather evidence on the role played by environmental cofactors, and their interplay with genomics, in the etiology of complex psychiatric and neurological disorders. The review by Robinson and Bergen highlighted that the investigation of gene-by-environment interaction in both schizophrenia and bipolar disorder has been often hindered by methodological problems including inadequate statistical power. However, they also showed that the advent of large longitudinal cohort studies is revealing genetic interactions with environmental exposures that contribute to risk for these disorders, although more data are available for SZ than for BD, and the extent to which these account for the total risk from environmental sources remains unknown (Robinson and Bergen). In accordance with this line of evidence, the mini review by Chen et al. showed that epigenetic mechanisms (typically activated by environmental triggers) including DNA methylation, ncRNA transcriptional regulation and histone modification, play an important part in the pathogenesis of schizophrenia suggesting a possible role of these markers for diagnostic and prognostic purposes. The remaining set of articles provided insights on the role of genetic and environmental determinants in neurodegenerative disorders such as Alzheimer's disease (Contini et al.; Upadhya et al.), Parkinson's disease (Vishweswaraiah et al.), and multiple sclerosis (Li et al.). Contini et al. showed that the salivary protein profile of patients with Alzheimer's disease was characterized by significantly higher levels of some multifaceted proteins and peptides that were either specific to the oral cavity or also expressed in other body districts. These included peptides involved in the homeostasis of the oral cavity; proteins with a neuroprotective role, such as S100A8, S100A9, and their glutathionylated and nitrosylated proteoforms, cystatin B and glutathionylated and dimeric derivatives, and proteins with antimicrobial activity, such as α-defensins, cystatins A and B, histatin 1, statherin, and thymosin β4, the latter with a neuroprotective role at the level of microglia. These findings suggest that patients with Alzheimer's disease enact compensatory mechanisms detectable at the oral level that counteract the neurodegenerative processes. Further, Upadhya et al. tested how genetic risk could affect depression onset in individuals with late-onset Alzheimer's Disease. Using polygenic risk scores (PRS) derived from the summary statistics of the most recent genome-wide association study (GWAS) of Major Depressive Disorder (MDD), the authors found that the model incorporating PRS with baseline age, sex, education, and APOEε4 allele count predicted depression onset with moderate accuracy (Upadhya et al.). In Parkinson's disease Vishweswaraiah et al. described the integration of metabolomics and epigenetics (genome-wide DNA methylation; epimetabolomics) to profile the frontal lobe in patients who died from Parkinson's disease in comparison with age-, and sex-matched controls. The authors identified 48 metabolites, 4,313 differentially methylated sites, and increased DNA methylation age in the primary motor cortex of people deceased for Parkinson's disease (Vishweswaraiah et al.). Importantly, primary bile acid biosynthesis was the major biochemical pathway identified as perturbed in the frontal lobe, with the metabolite taurine positively correlated with CpG cg14286187 (Vishweswaraiah et al.). Finally, Li et al. analyzed a GWAS dataset of 9,772 cases of multiple sclerosis and 17,376 healthy controls of European descent using gene-based tests. The authors identified 28 shared genes and of these, ten of the 28 genes were significantly differentially expressed in the multiple sclerosis case-control gene expression omnibus (GEO) database. Interestingly, GALC and HLA-DOB showed the most prominent differences in gene expression (two- and three-fold, respectively) between patients and healthy controls (Li et al.).

In summary, this Research Topic showed that there are substantial advancements in the comprehension of environmental contribution to the liability of complex disorders. It appears crucial the development of novel analytical approaches that can address the challenges of identifying the magnitude of its impact in large scale datasets.

## Author contributions

MM, DF, GF, and CZ took part in drafting the article, revising it critically for important intellectual content, gave final approval of the version to be published, and agree to be accountable for all aspects of the work.

## Funding

This paper was partly funded by Fondo Integrativo per la Ricerca (FIR)-2020 granted to MM.

## Conflict of interest

The authors declare that the research was conducted in the absence of any commercial or financial relationships that could be construed as a potential conflict of interest.

## Publisher's note

All claims expressed in this article are solely those of the authors and do not necessarily represent those of their affiliated organizations, or those of the publisher, the editors and the reviewers. Any product that may be evaluated in this article, or claim that may be made by its manufacturer, is not guaranteed or endorsed by the publisher.

## References

[B1] Gibson G. (2009). Decanalization and the origin of complex disease. Nat. Rev. Genet.[[Inline Image]] 10, 134–140. 10.1038/nrg2502 19119265

[B2] Hemminki K. Lorenzo Bermejo J. Försti A. (2006). The balance between heritable and environmental aetiology of human disease. Nat. Rev. Genet. 7, 958–965. 10.1038/nrg2009 17139327

[B3] Hu X. Walker D. I. Liang Y. Smith M. R. Orr M. L. Juran B. D.. (2021). A scalable workflow to characterize the human exposome. Nat. Commun. 12, 5575. 10.1038/s41467-021-25840-9 34552080 PMC8458492

[B4] Plomin R. von Stumm S. (2022). Polygenic scores: prediction versus explanation. Mol. Psychiatry 27, 49–52. 10.1038/s41380-021-01348-y 34686768 PMC8960389

[B5] Plomin R. Haworth C. M. A. Davis O. S. P. (2009). Common disorders are quantitative traits. Nat. Rev. Genet. 10, 872–878. 10.1038/nrg2670 19859063

[B6] Pries L.-K. Lage-Castellanos A. Delespaul P. Kenis G. Luykx J. J. Lin B. D.. (2019). Estimating exposome score for schizophrenia using predictive modeling approach in two independent samples: the results from the EUGEI study. Schizophr. Bull. 45, 960–965. 10.1093/schbul/sbz054 31508804 PMC6737483

[B7] Tenesa A. Haley C. S. (2013). The heritability of human disease: estimation, uses and abuses. Nat. Rev. Genet. 14, 139–149. 10.1038/nrg3377 23329114

[B8] Zhang H. Khan A. Kushner S. A. Rzhetsky A. (2022). Dissecting schizophrenia phenotypic variation: the contribution of genetic variation, environmental exposures, and gene–environment interactions. Schizophrenia 8, 1–5. 10.1038/s41537-022-00257-5 35853906 PMC9261082

